# Unraveling protein catalysis through neutron diffraction

**DOI:** 10.1063/4.0001110

**Published:** 2025-10-27

**Authors:** Dean A. Myles

**Affiliations:** Oak Ridge National Laboratory, Oak Ridge, TN, USA

## Abstract

Neutron diffraction provides a sophisticated, non-destructive means for the the atomic-resolution analysis of individual hydrogen atoms in enzymes. Here we describe how the precise location of protein and water hydrogen atoms using neutron diffraction provides a more complete description of the atomic and electronic structures of proteins, enabling key questions concerning enzyme reaction mechanisms, molecular recognition and binding and protein-water interactions to be addressed. We will briefly describe the capabilities that are available for neutron- based structural biology at ORNL, with special focus on the current upgrades and development of new instrumentation and software for neutron protein crystallographic analysis.

